# Socio-economic factors, mood, primary care utilization, and quality of life as predictors of intervention cessation and chronic stress in a type 2 diabetes prevention intervention (PREVIEW Study)

**DOI:** 10.1186/s12889-023-16569-9

**Published:** 2023-08-30

**Authors:** Maija Huttunen-Lenz, Anne Raben, Tanja Adam, Ian Macdonald, Moira A. Taylor, Gareth Stratton, Kelly Mackintosh, J. Alfredo Martinez, Teodora Handjieva-Darlenska, Georgi Assenov Bogdanov, Sally D. Poppitt, Marta P. Silvestre, Mikael Fogelholm, Elli Jalo, Jennie Brand-Miller, Roslyn Muirhead, Wolfgang Schlicht

**Affiliations:** 1 Institute of Nursing Science, University of Education Schwäbisch Gmünd, Oberbettringerstraße 200, 73525 Schwäbisch Gmünd, Germany; 2 Department of Nutrition, Exercise and Sports, University of Copenhagen, 1958 Frederiksberg, Denmark; 3 Clinical Research, Copenhagen University Hospital – Steno Diabetes Center Copenhagen, Herlev, Denmark; 4 Department of Nutrition and Movement Sciences, NUTRIM, School of Nutrition and Translational Research in Metabolism, Maastricht University, Maastricht, the Netherlands; 5 MRC/ARUK Centre for Musculoskeletal Ageing Research, National Institute for Health Research (NIHR) Nottingham Biomedical Research Centre, University of Nottingham, School of Life Sciences, Nottingham, NG7 2UH UK; 6 Nestle Institute of Health Sciences, Nestle Research, Route du Jorat 57, 1000 Lausanne 26, CH Switzerland; 7 University of Nottingham, School of Life Sciences, Nottingham, NG7 2UH UK; 8 Sport and Exercise Sciences, Swansea University, Swansea, West Glamorgan UK; 9 Applied Sports, Technology, Exercise and Medicine Research Centre, Swansea University, Swansea, West Glamorgan UK; 10 Department of Medicine and Endocrinology, University of Valladolid, Valladolid, Spain; 11 CIBER Fisiopatología Obesidad Y Nutrición (CIBERobn), Instituto de Salud Carlos III, IMDEAfood Madrid, 28029 Madrid, Spain; 12 Department of Pharmacology and Toxicology, Medical University of Sofia, Sofia, 1000 Bulgaria; 13 Department of Medicine, University of Auckland, Human Nutrition Unit, School of Biological Sciences, Auckland, 1024 New Zealand; 14 Human Nutrition Unit, School of Biological Sciences, University of Auckland, Auckland, 1024 New Zealand; 15 Nutrition & Metabolism, CINTESIS, NOVA Medical School, Faculdade de Ciências Médicas, NMS, FCM, Universidade NOVA de Lisboa, Lisbon, Portugal; 16 Department of Food and Nutrition, University of Helsinki, 00014 Helsinki, Finland; 17 School of Life and Environmental Sciences and Charles Perkins Centre, University of Sydney, Camperdown, NSW 2006 Australia; 18 Department of Exercise and Health Sciences, University of Stuttgart, 70569 Stuttgart, Germany

**Keywords:** Diabetes Mellitus, Overweight, Quality of Life, Lifestyle, Adherence, Prevention, Drop out, Stress, Health Behaviors

## Abstract

**Background:**

Sedentary lifestyle and unhealthy diet combined with overweight are risk factors for type 2 diabetes (T2D). Lifestyle interventions with weight-loss are effective in T2D-prevention, but unsuccessful completion and chronic stress may hinder efficacy. Determinants of chronic stress and premature cessation at the start of the 3-year PREVIEW study were examined.

**Methods:**

Baseline Quality of Life (QoL), social support, primary care utilization, and mood were examined as predictors of intervention cessation and chronic stress for participants aged 25 to 70 with prediabetes (*n* = 2,220). Moderating effects of sex and socio-economic status (SES) and independence of predictor variables of BMI were tested.

**Results:**

Participants with children, women, and higher SES quitted intervention earlier than those without children, lower SES, and men. Lower QoL, lack of family support, and primary care utilization were associated with cessation. Lower QoL and higher mood disturbances were associated with chronic stress. Predictor variables were independent (*p* ≤ .001) from BMI, but moderated by sex and SES.

**Conclusions:**

Policy-based strategy in public health should consider how preventive interventions may better accommodate different individual states and life situations, which could influence intervention completion. Intervention designs should enable in-built flexibility in delivery enabling response to individual needs.

**Trial registration:**

ClinicalTrials.gov Identifier: NCT01777893.

## Introduction

Globally, Type 2 Diabetes (T2D) is a cause of a major disease burden [1], with obesity and sedentary lifestyle as key risk factors in development and progression of T2D [2]. Despite individual-centered public health efforts (i.e. lifestyle interventions), T2D prevalence in high-income economies such as those in western Europe is at estimated 8.5% and still increasing [1, 3, 4], with those persons in lower socio-economic status (SES) especially affected [5–7]. Consequences of T2D can be serious, encompassing physical and psychological aspects of health, thus adversely impacting on individuals’ Quality of Life (QoL) [8, 9]. In T2D-prevention, lifestyle interventions supporting weight-loss and weight-loss maintenance have potential to improve health related outcomes [10–13], and, consequently, QoL [14, 15].

Despite the potential benefits of lifestyle interventions in T2D-prevention, premature intervention cessation and stress are leading to sub-optimal intervention benefits [13, 16–18]. Therefore, it is not enough to identify those individuals, who are benefiting from interventions (i.e. “successful achievers”) [18], but also to identify those pathways that influence success of weight-loss and weight-maintenance interventions in T2D-prevention [19, 20].

Premature intervention cessation is the result of a complex interaction between intervention inputs, individuals, and context variables [19, 21, 22]. The question is known as “whiches conundrum” asked by King [23]: *“Which intervention, for which people, under which circumstances?”* While personality traits (e.g. neuroticism, extraversion) do not appear to be associated with intervention cessation in T2D-prevention [22], factors such as higher baseline body mass index (BMI), younger age, employment or study, hesitancy about the efficiency of lifestyle changes, have been associated with intervention cessation [16, 24–29]. There is no consensus about the influence of factors such as low mood on intervention cessation [22, 30]. This may likely be due to the fact, that associations of mood and treatment cessation are moderated by other variables.

Identification of pathways to successful weight-loss maintenance is challenging owing to the interconnectivity and interchangeability between factors, in where factors can have both direct and indirect influence on the outcomes [20, 31, 32]. Precisely because of the partly contradictory findings [33, 34], an improved understanding of the interactions between different factors would enable more targeted lifestyle interventions supporting weight-loss and weight-loss maintenance for individuals with prediabetes. While a considerable body of literature has examined factors associated with successful weight-loss maintenance [34, 35], there is limited evidence from large scale studies examining complex pathways associated with intervention cessation and chronic stress in T2D-prevention.

In this study, health-related QoL, social support, use of primary care, and mood at the start of a lifestyle intervention were examined. It was hypothesized that these variables function influence intervention cessation and chronic stress, both of which are associated with less favorable weight-loss and weight-loss maintenance outcomes [13, 33, 36]. Furthermore, it was examined whether sex and SES moderated relationships between health-related QoL, social support, use of primary care, and mood as predictors of intervention cessation and chronic stress.

Higher risk of adverse consequences from T2D has been associated, for example, with sex (men), non-Caucasian ethnicity, and lower SES, which, in turn, are associated with lower likelihood of enrolment and higher likelihood of intervention cessation [37–41]. Sex has been indicated as a potential moderator for variables such as mood, chronic stress, and eating restraint during weight-loss and weight maintenance [18, 42]. Although social support has been associated with positive weight-loss outcomes especially among women, overall role of social support in T2D-prevention is less well understood [29]. Further, while men are less likely to participate and attend regularly in lifestyle interventions, role of participant’s sex in moderating relationships between different predictor variables and intervention cessation and chronic stress during weight-loss and weight loss maintenance is less clear [26, 27, 43].

Lower SES (measured as income) and higher chronic stress have been associated with worse weight-loss outcomes for both men and women and with overall lower QoL [18, 44–47], as well as with intervention cessation [18, 45]. In addition, associations between weight and SES (measured as level of education and income), appear, at least partially, mediated by sex [5, 48, 49]. For women, higher SES has been associated with lower likelihood of obesity but similar association has not been observed between men in lower and higher SES [50].

Beyond premature intervention cessation, chronic stress has been identified as one of the key factors hindering weight-loss and weight-loss maintenance efforts in lifestyle interventions [18, 36]. Stress, a physiological and/or psychological response to perceived internal or external stressors can be seen as adaptive (short-term) or harmful (long-term) [51]. Stressors, i.e. events or conditions that lead to physical or psychological stress, can be dependent on individual’s life situation, SES, and ethnic group membership [51–53]. Especially chronic stress influences individuals’ biological systems negatively, which, consecutively, can have negative influences on daily functioning, cognitive capacities, and health [52]. Stress is also associated with poorer health behaviors, including poorer dietary choices and physical inactivity [36, 54], thus increasing the risk of weight-gain [32, 55]. Subsequently, higher perceived chronic stress can be counterproductive in lifestyle modification interventions aiming for weight-loss and weight-loss maintenance [18, 32]. Lower SES has been associated with higher stress and increased weight-gain especially among men [31].

In these secondary analyses, data were analyzed from a group-based T2D-prevention intervention PREVIEW (PREVention of diabetes through lifestyle Intervention and population studies in Europe and around the World) with weight-loss and weight-loss maintenance phases [13]. Here at the baseline of the PREVIEW intervention, it was examined whether the QoL, social support, primary care utilization, and mood at the PREVIEW intervention baseline were associated, firstly, with intervention cessation and, secondly, with chronic stress. We hypothesized that lower QoL and lower social support, as well as higher primary care use and mood disturbances would predict higher likelihood of intervention cessation and higher chronic stress. Thirdly, we examined if QoL, social support, primary care utilization, and mood were independent predictors of intervention cessation and chronic stress over and above baseline BMI. We hypothesized that QoL, social support, primary care utilization, and mood would predict intervention cessation independently from BMI. Fourthly, we examined whether sex and SES moderated relationships between predictor variables (i.e. QoL, social support, primary care utilization, mood) and intervention cessation and chronic stress. Here, degree of education was used as an indicator of SES [56, 57].

## Methods

### PREVIEW Intervention

**Fig. 1:**
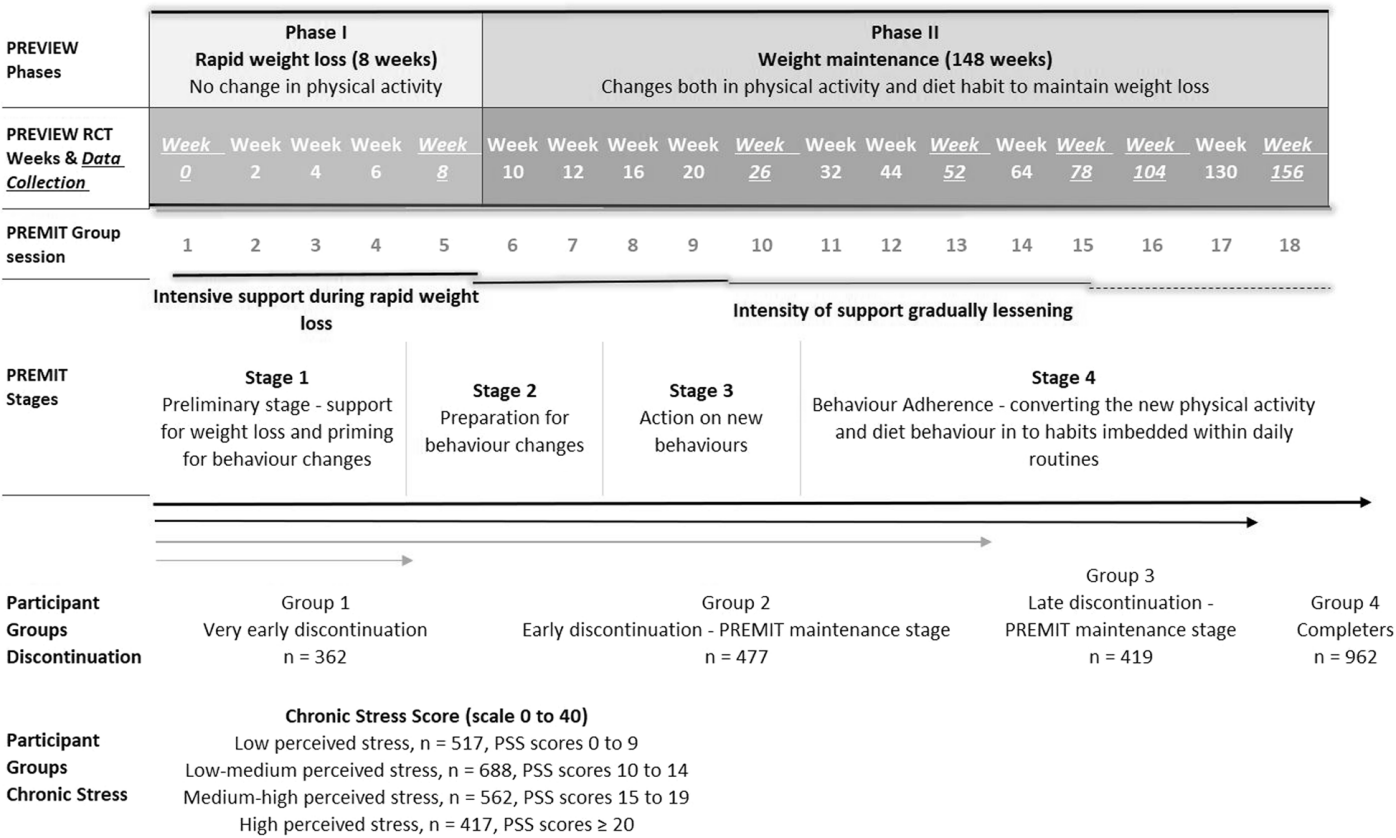
PREVIEW Study timeline

The PREVIEW intervention was a 36-month randomized controlled trial (RCT) (Fig. 1), comprising a 2-month weight-loss phase for all participants and a 34-month weight maintenance phase for those who lost ≥ 8% of their baseline body mass during the initial weight-loss phase [58]. During the weight-loss phase, rapid weight-loss was supported through use of a low energy diet (Cambridge Weight Plan™). Participants were not expected to change physical activity (PA) habits during this phase. For the weight maintenance phase, participants were randomized to four different intervention arms, in a 2 × 2 factorial design, which covered two dietary and two PA programs to maintain the achieved ≥ 8% weight-loss (diet programs: higher protein, moderate carbohydrate, low glycemic index vs. moderate protein, higher carbohydrate, medium glycemic index diet; physical activity programs: high-intensity PA vs. moderate-intensity PA). [58]. A behavior modification intervention PREMIT (PREview behavior Modification Intervention Toolbox) supported participants to modify their diet and PA behaviors to achieve and maintain weight-loss during the PREVIEW intervention [59]. Detailed information about the design and methods of the PREVIEW intervention including sample size calculations and detailed participant inclusion and exclusion criteria can be found in Fogelholm et al. [58].

### PREMIT behavior modification intervention

PREMIT was a stage-based and theory-orientated intervention designed as an integral part of the PREVIEW intervention, but not specific to PREVIEW RCT arm. All participants received the same behavioral support. PREMIT had 4 stages: preliminary (stage 1); preparation (stage 2); action (stage 3); and maintenance (stage 4). PREMIT was delivered over 18 group sessions with 10–20 participants in a group (Fig. 1) [59].

Different behavioral techniques were implemented to support participants in performing and maintaining the new diet and PA behaviors. Behavioral techniques such as information, planning for social support, modelling behaviors, and goal setting were used to influence behavioral determinants including self-efficacy, social support, and action planning. PREMIT was designed so that participants could apply the learned techniques in real life situations at home, at work, and in community environment. Intensity of support was reduced gradually when it was assumed that the new behaviors would have been imbedded into daily routines [59].

### Participants

Participants were recruited from eight study sites; Copenhagen (Denmark), Helsinki (Finland), Nottingham (United Kingdom), Sydney (Australia), Maastricht (The Netherlands), Sofia (Bulgaria), Navarra (Spain), Auckland (New Zealand). Individuals irrespective of sex aged 25 to 70 years of age with overweight or obesity (BMI ≥ 25 kg/m^2^) and confirmed prediabetes as either impaired fasting plasma glucose and/or an impaired 2-h oral glucose tolerance test were eligible to participate [60]. Main exclusion criteria initially were T2D and any known illness or medication that had an influence on compliance e.g. with PA-program. At the screening stage the main exclusion criteria were normoglycemia or confirmed T2D, and findings of any condition, illness, or medication that could influence compliance with the diet or PA-program [58].

Participant were recruited from June 2013 to April 2015 through referrals from primary and occupational healthcare providers and from advertising both in print and visual media. Those interested in participation were pre-screened prior to an invitation for a full screening. To ensure consistency in recruitment, the same procedures, in local languages, were followed in each study site. The study protocol was reviewed by the relevant local research ethics committee in each study site. Participants were required to provide signed informed consent prior to study enrolment [58].

### Estimation of socio-economic status (degree of education)

Participants’ SES was estimated using the highest degree of education achieved [56, 57, 61]. It was recognized that SES is a multifaceted construct including occupation, education degree, and available income over life course [7, 57]. However, degree of education has been shown an important determinant of social and health inequalities in T2D [5, 49]. More sophisticated methods such as Blinder-Oaxaca decomposition were not used to estimate SES due to suboptimal occupational data and difficulties of comparing available incomes across the intervention sites of different countries taking part in PREVIEW intervention [6].

### Data collection and measurements

Data from anthropometric (weight and height), social-cognitive (social support, mood, chronic stress, QoL, primary care utilization, and socio-demographic characteristics) were collected at the intervention baseline. Data for intervention cessation were collected at each data assessment point (Fig. 1). All social-cognitive data collection was done in local languages. If required, questionnaires were translated into local languages using standard practice of translation and back-translation.

#### Body mass and height

Weight and height were measured lightly clad without shoes and BMI was calculated as (weight/height^2^).

#### Socio-demographic characters

Data were collected using The European Social Survey and International Social Survey [62] and included variables such as age, sex, and level of education.

#### Social support

Based on the scale developed by Sallis et al. [63], perceived social support by family and friends for healthy diet and PA behaviors was assessed. Participants were asked, for example, whether family and/or friends exercised with them or encouraged eating healthy foods. For each question participants were asked to assess the frequency of social support on the scale “1” (*none*) to “5” (*very often*), separately for family and friends. Option “does not apply” could also be chosen, which, for analyses, was recoded as “1” (none). Lower values indicated less social support.

Factor analysis with Principal Components method and Varimax rotation with Eigenvalue 2 as a cut of point to limit the number of domains was performed. The suggested factor solution explained 54.7% of the total variance. Both family and friend variables loaded to one domain for diet encouragement (Eigenvalue 3.3) and diet discouragement (Eigenvalue 2.7). However, as the family and friends’ variables did not correlate closely, both dimensions were divided in two domains: encouragement healthy diet family (5 questions) and friends (5 questions); discouragement healthy diet family (5 questions); and friends (5 questions). Similarly, for PA support (rewards and punishment s) all variables loaded in one dimension (Eigenvalue 2.2) but were divided in two domains: family (3 questions); and friends (3 questions). For PA participation, variables loaded to two different domains: PA participation family (Eigenvalue 5.0, 10 questions); and PA participation friends (Eigenvalue 12.0, 10 questions).

Cronbach’s Alphas were calculated for each domain. For diet domain Cronbach’s Alphas ranged from *α* = 0.88 (family engagement) to *α* = 0.76 (friends’ discouragement). For PA domain Cronbach’s Alphas ranged from *α* = 0.91 (friends’ participation) to *α* = 0.44 (family support). As the Cronbach’s Alphas indicated low reliability for both family (*α* = 0.44) and friends (*α* = 0.58) PA support, these domains were excluded from further analyses. The following six domains were included in the analyses; (i) family diet encouragement and (ii) discouragement, (iii) friends diet encouragement and (iv) discouragement, (v) family PA participation, and (vi) friends PA participation.

#### Quality of life

QoL was assessed using the World Health Organization [64] instrument, which included 26 questions and five domains: Physical Health (7 question); Psychological Health (6 questions); Social Relationships (3 questions); Environment (8 questions); Overall Quality of Life (2 questions). Participants were asked to rate each question, for example, “Are you able to accept your bodily appearance?” on the scale “1” (*not at all*) to “5” (*completely*). Mean score was calculated for each domain and transformed to be comparable with the WHO-QoL-100 [65, 66]. Higher scores indicated better perceived QoL. Cronbach’s Alphas were calculated for each domain and ranged from *α* = 0.59 for Overall QoL to *α* = 0.79 for Physical Health. Due to low reliability, the domain for Overall QoL was excluded from further analyses.

#### Mood states

Mood states were assessed using the Profile Of Mood States Questionnaire (POMS) [67], which consisted 65 statements of feelings, divided in 6 domains: tension; depression; anger; fatigue; vigor; and confusion. For each feeling, participants were asked to rate whether they had experienced it during the last week from “0” (*not at all*) to “4” (*extremely*). Sum score of mood disturbance (maximum 200) was calculated by adding domains scores of tension, depression, anger, fatigue, and confusion together before subtracting the vigor domain scores. Lower scores indicated more stable mood. Cronbach’s Alphas for the different domains ranged from *α* = 0.77 for confusion to* α* = 0.93 for depression.

#### Chronic stress

Chronic stress was assessed by the perceived stress Scale (PSS) [68]. The PSS is an instrument measuring the extent to which individuals perceive situations in their life as stressful. Participants were asked to rate 10 questions about feelings and thoughts related to life situations during the last month on scale “0” (*never*) to “4” (*very often*) during the last month. Sum-score was calculated with higher scores indicating higher perceived stress with a Cronbach’s Alpha α = 0.83.

#### Primary healthcare utilization

A healthcare utilization questionnaire was developed for the PREVIEW intervention to assess utilization of healthcare services especially within primary care, based on existing questionnaires such as Schweikert et al. [69]. Participants were asked about frequency of contact with healthcare practitioners during the last three months, including doctors, nurses, dieticians, and physiotherapists. In addition, participants were asked about use of medication during the last three months (both prescription and non-prescription), and whether they had spent money for physical activity pursuits. The following areas of healthcare utilization during the last three months were included in the analyses; visits to medical or nurse practitioners, visits to other healthcare practitioners, renewal of prescriptions, receiving advice about diet or PA, medication and supplement use, money spent on PA activities.

### Statistical analyses

Analyses were based on the 2,220 participants with height and body mass data at the start of the study commencement. For the purposes of this study, participants were divided in four groups separately for both outcome variables (cessation and chronic stress, Fig. 1). For cessation, intervention theory was used to guide decision for cessation cut-off points (Figure); group 1 – “very early cessation”—participants who did not achieve the weight-loss target and dropped out by week 8 (*n* = 362); group 2 – “early cessation”—participants who dropped out between weeks 8 and 52, (*n* = 477); group 3 – “late cessation”—participants who dropped out between weeks 52 and 156 (*n* = 419); group 4 – “completers”—participants who completed the intervention (*n* = 962). For chronic stress, the groups, depending on their stress scores were; group 1 – “low chronic stress” (*n* = 524, PSS scores “0 to 9”); group 2 – “low-medium chronic stress” (*n* = 694, PSS scores “10 to 14”); group 3 – “medium–high chronic stress” (*n* = 565, PSS scores “15 to 19”); group 4 – “high chronic stress” (*n* = 437, PSS scores ≥ 20). Descriptive statistical methods were used to describe participant characteristics.

Data were checked for missing values. For continuous variables of social support, intentions, QoL, mood, and stress missing values were imputed using automatic method with 5000 case and four parameter draws. For the categorial variable of “primary healthcare utilization” missing values were conservatively recoded as negative values i.e. no healthcare service use or money spent for PA. Data were inspected for outliers and normality. All values were within the expected ranges. Multivariate outliers for continuous variables (QoL, social support, and mood) were examined using Mahalanobis distance with probability of *p* < 0.001, and 36 cases were identified as a multivariate outlier, but not removed as all values were within expected ranges. Due to the way POMS scale scores were calculated, 27.1% of the scores were negative and a constant of + 28 was applied to all scores to ensure a minimum score of 0. Although none of the continuous variables were normally distributed, no data transformations were undertaken as this was not necessary for the selected statistical analysis methods [70].

Chi-Square tests were used examine differences in demographic characteristics between the different outcome categories for drop out and chronic stress using standardized residual of ≤  ± 2.24 ≥ as a cut-off point for significance. Three multinomial logistic regressions were performed for both outcome variables; (1) time of cessation with completers as a reference category, and (2) chronic stress with low chronic stress as a reference category. For both outcomes (time of cessation, chronic stress), the first model tested overall significance with age, degree of education, mood, healthcare utilization, QoL, and social support as predictor variables.

The second model examined whether variables that emerged as significant predictors in the first model predict intervention cessation and chronic stress independently from BMI. For the second model sequential approach was used. First, a logistic regression (lr_a) was calculated with intervention cessation/chronic stress as the dependent variable and BMI as the predictor variable. Second, a logistic regression (lr_b) was calculated with BMI and significant predictors from the model one. Significance of the *χ*2 change between the models was calculated as (*χ*2 = lr_b – lr_a = x, df = lr_b – lr_a = x, *χ*2 (x) = x).

The third model tested interaction effects with sex (two categories) and degree of education (five categories) as moderating variables. When analyses indicated moderation but more than one category of the moderator appeared to be significant (e.g. both sexes emerged as potentially significant moderators), the following formula was used to determinate whether there was significant difference between the categories’ regression coefficients with z ≤  ± 2.24 ≥ used as a cut-off point for significant differences. As the variable education had five categories, the lowest significant degree of education was used as a reference category. For the variable sex was dichotomous, no reference category was needed.

$$Z=\frac{b1-b2}{\sqrt{SE{b}_{1}^{2}}+SE{b}_{2}^{2}}$$

Due to multiple testing, the Bonferroni adjusted p-value of *p* ≤ 0.012 was used for both outcome variables (cessation, chronic stress). Sensitivity analyses were performed using both the original dataset and dataset with multivariate outliers removed. As no significant differences were found, results are reported only for the imputed dataset without removing outliers. All the analyses were performed with the IBM SPSS® statistical program v27.

## Results

Participants and predictor variables characteristics are shown in Tables 1 and 2. Chi-square tests between points of cessation, stress, and social-demographic characteristics were performed and summarized in Table 3.

**Table 1:** Baseline participants demographic characteristics for all participants and separated by point of cessation and chronic stress at the beginning of the PREVIEW intervention

Participants’ demographic characteristics	All *n* = 2220	PREVIEW cessation				Chronic stress			
		**Very early *n* = 362**	**Early *n* = 477**	**Late *n* = 419**	**Completers *n* = 962**	**Low *n* = 524**	**Low-medium *n* = 694**	**High- medium *n* = 565**	**High *n* = 437**
***Age (m* ± *sd)***	51.6 ± 11.6	47.6 ± 12.5	48.4 ± 12.1	51.1 ± 11.2	54.8 ± 10.1	55.5 ± 10.4	52.9 ± 11.0	49.2 ± 11.7	47.8 ± 11.8
***Sex (%)***									
Men	720 (32.4%)	96 (26.5%)	155 (32.5%)	126 (30.1%)	343 (35.7%)	224 (42.7%)	240 (34.6%)	155 (27.4%)	101 (23.1%)
Women	1500 (67.6%)	266 (73.5%)	322 (67.5%)	293 (69.9%)	619 (64.3%)	300 (57.3%)	454 (65.4%)	410 (72.6%)	336 (76.9%)
***BMI (m* ± *sd)***	35.4 ± 6.6	36.4 ± 7.9	37.0 ± 6.9	36.8 ± 6.6	33.5 ± 5.3	34.3 ± 5.4	34.6 ± 5.9	35.8 ± 6.8	37.2 ± 8.0
***Degree of education (%)***									
Up to secondary education	373 (16.8%)	75 (20.7%)	79 (16.6%)	86 (20.5%)	133 (13.8%)	74 (14.1%)	131 (18.9%)	78 (13.8%)	90 (20.6%)
Secondary vocational education	389 (17.5%)	61 (16.9%)	100 (21.0%)	64 (15.3%)	164 (17.0%)	101 (19.3%)	106 (15.3%)	105 (18.6%)	77 (17.6%)
Higher vocational education	367 (16.5%)	46 (12.7%)	65 (13.6%)	70 (16.7%)	186 (19.3%)	109 (20.8%)	123 (17.7%)	82 (14.5%)	53 (12.1%)
University education	835 (37.6%)	134 (37.0%)	176 (36.9%)	161 (38.4%)	364 (37.8%)	176 (33.6%)	255 (36.7%)	242 (42.8%)	162 (37.1%)
Other	256 (11.5%)	46 (12.7%)	57 (11.9%)	38 (9.1%)	115 (12.0%)	64 (12.2%)	79 (11.4%)	58 (10.3%)	55 (12.6%)
***Ethnicity***									
Caucasian	1944 (87.6%)	279 (77.1%)	429 (89.9%)	345 (82.3%)	891 (92.6%)	484 (92.4%)	622 (89.6%)	485 (85.8%)	353 (80.8%)
Other	276 (12.4%)	83 (22.9%)	48 (10.1%)	74 (17.7%)	71 (7.4%)	40 (7.6%)	72 (10.4%)	80 (14.2%)	84 (19.2%)
***Marital status***									
Married or Civil Partnership	1502 (67.7%)	212 (58.6%)	309 (64.8%)	267 (63.7%)	714 (74.2%)	366 (69.8%)	484 (69.7%)	385 (68.1%)	267 (61.1%)
Divorced, widowed, separated	354 (15.9%)	73 (20.2%)	74 (15.5%)	73 (17.4%)	134 (13.9%)	99 (18.9%)	105 (15.1%)	76 (13.5%)	74 (16.9%)
Single or other	364 (16.4%)	77 (21.3%)	94 (19.7%)	79 (18.9%)	114 (11.9%)	59 (11.3%)	105 (15.1%)	104 (18.4%)	96 (22.0%)
***Household – Living with others***									
One adult	447 (20.1%)	84 (23.2%)	98 (20.5%)	88 (21.0%)	177 (18.4%)	109 (20.8%)	129 (18.6%)	109 (19.3%)	100 (22.9%)
Two adults	922 (41.5%)	120 (33.1%)	182 (38.2%)	167 (39.9%)	453 (47.1%)	261 (49.8%)	310 (44.7%)	218 (38.6%)	133 (30.4%)
Three or more adults	340 (15.3%)	51 (14.1%)	76 (15.9%)	64 (15.3%)	149 (15.5%)	68 (13.0%)	113 (16.3%)	85 (15.0%)	74 (16.9%)
One adult and at least one child	48 (2.2%)	18 (5.0%)	14 (2.9%)	11 (2.6%)	5 (0.5%)	-	16 (2.3%)	20 (3.5%)	11 (2.5%)
Two adults and at least one child	397 (17.9%)	73 (20.2%)	90 (18.9%)	76 (18.1%)	158 (16.4%)	74 (14.1%)	111 (16.0%)	117 (20.7%)	95 (21.7%)
Three or more adults at least one child	66 (3.0%)	16 (4.4%)	17 (3.6%)	13 (3.1%)	20 (2.1%)	11 (2.1%)	15 (2.2%)	16 (2.8%)	24 (5.5%)
***Employment***									
In Study or Employment (Regardless of hours)	1429 (64.4%)	226 (62.4%)	315 (66.0%)	298 (71.1%)	590 (61.3%)	317 (60.5%)	463 (66.7%)	392 (69.4%)	257 (58.8%)
Not economically active (e.g. carer, unemployed, off sick)	230 (10.4%)	52 (14.4%)	56 (11.7%)	39 (9.3%)	83 (8.6%)	32 (6.1%)	60 (8.6%)	63 (11.2%)	75 (17.2%)
Retired	375 (16.9%)	47 (13.0%)	62 (13.0%)	57 (13.6%)	209 (21.7%)	126 (24.0%)	125 (18.0%)	71 (12.6%)	53 (12.1%)
Other	186 (8.4%)	37 (10.2%)	44 (9.2%)	25 (6.0%)	80 (8.3%)	49 (9.4%)	46 (6.6%)	39 (6.9%)	52 (11.9%)

Please note: Categories “other” include missing values; Cells with fewer than 5 participants not shown

**Table 2:** Descriptive statics for the variables of QoL, mood, social support, and healthcare utilisation for all participants and separated by point of cessation and chronic stress at the beginning of the PREVIEW intervention

		**PREVIEW cessation**				**Chronic stress**			
**Social-cognitive variables (Scale range) m ± sd Healthcare utilisation (Scale range) n / %**	**Participants All** ***n* = 2220**	**Very early** ***n* = 362**	**Early** ***n* = 477**	**Late** ***n* = 419**	**Completers** ***n* = 962**	**Low** ***n* = 524**	**Low-medium** ***n* = 694**	**High- medium** ***n* = 565**	**High** ***n* = 437**
***Social-cognitive variables***									
QoL Physical health (4 – 20)	15.1 ± 2.5	14.4 ± 2.6	14.7 ± 2.6	15.0 ± 2.5	15.6 ± 2.2	16.6 ± 1.9	15.8 ± 1.9	14.7 ± 2.1	12.8 ± 2.6
QoL Psychological (4 – 20)	14.0 ± 2.3	13.5 ± 2.4	13.7 ± 2.4	13.9 ± 2.3	14.4 ± 2.1	15.6 ± 1.7	14.6 ± 1.7	13.5 ± 1.9	11.9 ± 2.3
QoL Social relationships (4 – 20)	14.6 ± 2.8	14.1 ± 3.0	14.4 ± 2.9	14.4 ± 2.8	14.9 ± 2.6	15.7 ± 2.4	15.0 ± 2.4	14.2 ± 2.6	12.9 ± 3.2
QoL Environment (4 – 20)	15.2 ± 2.3	14.2 ± 2.3	14.7 ± 2.3	15.3 ± 2.2	15.9 ± 2.0	16.7 ± 1.7	15.7 ± 1.8	14.6 ± 2.0	13.4 ± 2.3
Chronic stress (0 – 40)	14.2 ± 6.3	16.5 ± 6.4	14.9 ± 6.3	14.2 ± 6.1	13.0 ± 6.0	26.7 ± 16.6	38.7 ± 18.0	54.3 ± 22.5	85.4 ± 33.9
Mood states (0 – 228)	49.0 ± 30.7	57.8 ± 34.1	52.4 ± 31.1	50.4 ± 31.9	43.5 ± 27.3	6.3 ± 1.0	6.3 ± 1.0	6.3 ± 1.0	6.1 ± 1.1
Social support—family encouragement diet (1 – 5)	2.7 ± 1.2	3.0 ± 1.2	2.8 ± 1.2	2.7 ± 1.1	2.6 ± 1.1	1.9 ± 0.9	2.1 ± 0.9	2.2 ± 1.0	2.4 ± 1.1
Social support—family discouragement diet (1 – 5)	2.1 ± 1.0	2.4 ± 1.1	2.2 ± 1.0	2.2 ± 1.0	1.9 ± 0.9	2.0 ± 0.8	2.1 ± 0.8	2.3 ± 0.9	2.4 ± 0.9
Social support—friends’ encouragement diet (1 – 5)	2.2 ± 0.9	2.4 ± 0.9	2.2 ± 0.9	2.2 ± 0.9	2.1 ± 0.8	1.8 ± 0.7	2.0 ± 0.7	2.1 ± 0.8	2.2 ± 0.9
Social support—friends’ discouragement diet (1 – 5)	2.0 ± 0.8	2.2 ± 0.9	2.1 ± 0.8	2.0 ± 0.8	1.9 ± 0.7	2.0 ± 0.9	2.1 ± 0.9	2.0 ± 0.8	2.1 ± 1.0
Social support—family participation PA (1 – 5)	2.0 ± 0.9	2.1 ± 0.9	1.9 ± 0.8	2.0 ± 0.9	2.1 ± 0.9	1.8 ± 0.8	1.9 ± 0.8	1.9 ± 0.8	1.9 ± 0.9
Social support—friends’ participation PA (1 – 5)	1.9 ± 0.8	2.0 ± 0.9	1.8 ± 0.8	1.9 ± 0.8	1.8 ± 0.8	16.6 ± 1.9	15.8 ± 1.9	14.7 ± 2.1	12.8 ± 2.6
***Primary healthcare utilisation during last 3 months***									
*Contact with GP*^a^* or NP*^a^									
No contact	995 (44.8%)	165 (45.6%)	231 (48.4%)	176 (42.0%)	423 (44.0%)	266 (50.8%)	320 (46.1%)	230 (40.7%)	179 (41.0%)
Once	566 (25.5%)	89 (24.6%)	115 (24.1%)	105 (25.1%)	257 (26.7%)	140 (26.7%)	180 (25.9%)	153 (27.1%)	93 (21.3%)
Twice	358 (16.1%)	54 (14.9%)	82 (17.2%)	75 (17.9%)	147 (15.3%)	69 (13.2%)	113 (16.3%)	104 (18.4%)	72 (16.5%)
Three or more times	301 (13.6%)	54 (14.9%)	49 (10.3%)	63 (15.0%)	135 (14.0%)	49 (9.4%)	81 (11.7%)	78 (13.8%)	93 (21.3%)
*Prescription renewal*									
Did not renew prescription	1669 (75.2%)	277 (76.5%)	374 (78.4%)	314 (74.9%)	704 (73.2%)	392 (74.8%)	525 (75.6%)	441 (78.1%)	311 (71.2%)
Renewed prescription	551 (24.8%)	85 (23.5%)	103 (21.6%)	105 (25.1%)	258 (26.8%)	132 (25.2%)	169 (24.4%)	124 (21.9%)	126 (28.8%)
*Diet or PA advice from healthcare professionals*									
Did not receive advice	1823 (82.1%)	309 (85.4%)	395 (82.8%)	333 (79.5%)	786 (81.7%)	449 (85.7%)	561 (80.8%)	455 (80.5%)	358 (81.9%)
Received advice	397 (17.9%)	53 (14.6%)	82 (17.2%)	86 (20.5%)	176 (18.3%)	75 (14.3%)	133 (19.2%)	110 (19.5%)	79 (18.1%)
*Referral to or contact with another specialist healthcare professional (any reason)*									
No referral or contact	1738 (78.3%)	279 (77.1%)	375 (78.6%)	342 (81.6%)	742 (77.1%)	440 (84.0%)	562 (81.0%)	428 (75.8%)	308 (70.5%)
Referral or contact	482 (21.7%)	83 (22.9%)	102 (21.4%)	77 (18.4%)	220 (22.9%)	84 (16.0%)	132 (19.0%)	137 (24.2%)	129 (29.5%)
*Money spend on PA activities*									
No spend	1112 (50.1%)	201 (55.5%)	286 (60.0%)	211 (50.4%)	414 (43.0%)	242 (46.2%)	319 (46.0%)	298 (52.7%)	253 (57.9%)
Spend	1108 (49.9%)	161 (44.5%)	191 (40.0%)	208 (49.6%)	548 (57.0%)	282 (53.8%)	375 (54.0%)	267 (47.3%)	184 (42.1%)
*Taking medication or supplements with or without prescription*									
No medication or supplements	885 (39.9%)	180 (49.7%)	221 (46.3%)	156 (37.2%)	328 (34.1%)	192 (36.6%)	276 (39.8%)	220 (38.9%)	197 (45.1%)
Medication or supplements	1335 (60.1%)	182 (50.3%)	256 (53.7%)	263 (62.8%)	634 (65.9%)	332 (63.4%)	418 (60.2%)	345 (61.1%)	240 (54.9%)

^a^*GP*  General practitioner, *NP*  Nursing practitioner

**Table 3:** Baseline group comparisons between demographic variables for cessation and chronic stress

Demographic characteristics	Overall *χ*^2^	PREVIEW cessation Significant Standard residuals (≥ ± 2.24)				Chronic stress Significant Standard residuals (≥ ± 2.24)			
	**Cessation Chronic stress**	**Very early** ***n* = 362**	**Early** ***n* = 477**	**Late** ***n* = 419**	**Completers** ***n* = 962**	**Low** ***n* = 524**	**Low-medium** ***n* = 694**	**High- medium** ***n* = 565**	**High** ***n* = 437**
***Ethnicity***	Cessation *χ*^2^(3) = 72.17, *p* < .001 Chronic stress *χ*^2^ (3) = 33.84, *p* < .001								
Other (i.e. non-Caucasian)		5.66	-	3.04	-4.44	-3.12	-	-	4.03
***Marital status***	Cessation *χ*^2^ (6) = 41.31, *p* < .001 Chronic stress *χ*^2^ (3) = 28.01, *p* < .001								
Married or Civil Partnership		-	-	-	2.47	-	-	-	-
Divorced, widowed, separated		-	-	-	-	-	-	-	-
Single or other		2.29	-	-	-3.48	-2.90	-	-	2.88
***Household*** ***Living with others***	Cessation *χ*^2^ (15) = 53.85, *p* < .001 Chronic stress *χ*^2^ (15) = 68.91, *p* < .001								
Two adults		-2.47	-	-	2.67	2.94	-	-	-3.60
One adult, at least one child		3.63	-	-	-3.46	-3.07	-	-	-
Two adults, at least one child		-	-	-	-	-	-	-	-
At least three adults, one child		-	-	-	-	-	-	-	3.05
***Employment***	Cessation *χ*^2^ (9) = 42.81,* p* < .001 Chronic stress *χ*^2^ (9) = 76.68, *p* < .001								
In Study or Employment (Regardless of hours)		2.37	-	-	-	-	-	-	-
Not economically active (e.g. carer, unemployed, off sick)		-	-	-	-	-3.02	-	-	4.42
Retired		-	-	-	3.65	3.98		-2.50	-2.42
Other		-	-	-	-	-	-	-	2.54

Non-Caucasian ethnicity, being single, and living in a household with at least one child were associated with lower likelihood of achieving the ≥ 8% weight-loss (cessation group 1) and high chronic stress (stress group 4). Higher likelihood of completing the PREVIEW intervention (cessation group 4) was associated with being married and living in a two-adult household. Retired participants were more likely not only to complete the intervention (cessation group 4), but also to report low chronic stress (stress group 4).

### Predictors of cessation

Multinomial logistic regression with cessation as an outcome variable and “age”, “degree of education”, “primary healthcare utilization”, “social support”, “moods”, and “QoL” as predictor variables indicated overall model significance (*χ*2 (66) = 347.8, *p* < 0.001) with good data fit (Pearson *χ*2 (6591) = 6651.9, *p* = 0.27). The following emerged as significant predictor variables; “QoL environment” (*χ*2 (3) = 52.7, *p* < 0.001), “family discouragement for diet” (*χ*2 (3) = 11.6, *p* = 0.009), “money spend on PA activities” (*χ*2 (3) = 15.2, *p* = 0.002), and “taking medication or supplements” (*χ*2 (3) = 18.4, *p* < 0.001).

Participants in very early (group 1) and early (group 2) cessation groups reported lower environmental QoL, while experiencing lower family support for diet changes. Participants in both, very early (group 1) and early (group 2) cessation groups, were also more likely to report not taking medication or supplements, while only participants in early cessation group (group 2) were less likely to spend money on PA (e. g. fitness offers) than those who completed the weight-maintenance phase. No variables were significantly associated with late cessation (group 3). Results with parameter estimates for variables associated with the different group memberships are summarized in Table 4.

**Table 4:** Parameter estimates for significant predictor variables for cessation compared to the PREVIEW study completers

	**β**	**Standard error**	**Wald**	**Df**	**Significance**	**Exp(β)**
VARIABLES ASSOCIATED WITH CESSATION						
Group 1 Very early cessation (Did not achieve weight-loss target)						
QoL Environment	-.25	.04	42.74	1	*p* < .001	.78
Social Support—Family discouragement diet	.30	.10	9.71	1	*p* = .002	1.35
Healthcare utilisation – Not taking medication or supplements	.50	.14	12.63	1	*p* < .001	1.66
Group 2 Early cessation (Drop-out by early PREMIT maintenance stage)						
QoL Environment	-.16	.04	21.28	1	*p* < .001	.85
No spend on PA activities during last 3 months	.47	.12	14.53	1	*p* < .001	1.60
Healthcare utilisation – Not taking medication or supplements	.42	.13	10.71	1	*p* = .001	1.51
SEX AS MODERATOR OF CESSATION						
Group 1 Very early cessation (Did not achieve weight-loss target)						
QoL Environment -						
Men	-.36	.08	20.73	1	*p* < .001	.70
Women	-.23	.05	26.76	1	*p* < .001	.79
*Between levels of up to secondary and secondary vocational z-score* = *3.92 significant*						
Family discouragement diet -						
Women	.31	.12	7.16	1	*p* = .007	1.37
Not taking medication or supplements -						
Women	.48	.17	8.21	1	*p* = .004	1.62
Group 2 Early cessation (Drop-out by early PREMIT maintenance stage)						
Not taking medication or supplements						
Men	.8	.20	11.5	1	*p* = .001	2.10
QoL Environment -						
Women	-.18	.04	17.68	1	*p* < .001	.84
Family discouragement diet –						
Women	.35	.11	10.15	1	*p* = .001	1.43
Group 3 Late Cessation (Drop out after early PREMIT maintenance stage)						
Family discouragement diet						
Women	.30	.11	6.80	1	*p* = .009	1.34
DEGREE OF EDUCATION AS MODERATOR OF CESSATION						
Group 1 Very early cessation (Did not achieve weight-loss target)						
QoL Environment -						
Up to secondary education	-.27	.10	7.77	1	*p* = .001	.77
Secondary vocational education	-.42	.10	16.32	1	*p* < .001	.66
University education	-.24	.06	14.91	1	*p* < .001	.79
*Between levels no significant differences*						
Family discouragement diet –Family discouragement diet –						
University education	.57	.16	12.68	1	*p* < .001	1.78
Group 2 Early cessation (Drop-out by early PREMIT maintenance stage)						
QoL Environment -						
University education	-.25	.06	19.48	1	*p* < .001	.78
Social Support—Family discouragement diet University education	.44	.15	8.67	1	*p* = .003	1.55
Group 3 Late cessation (Drop out after early PREMIT maintenance stage)						
Family discouragement diet –						
University education	.58	.15	14.00	1	*p* < .001	1.78

### Cessation – Independence of the significant predictors from BMI

Two logistic regressions were calculated with the cessation as dependent variable. The first model was calculated with “BMI” as the predictor variable (*χ*2 (3) = 149.5, *p* < 0.001, Goodness of fit Pearson *χ*2 (6589) = 6596.2, *p* = 0.45). The second model was calculated with “BMI”, “QoL environment”, “family discouragement for diet”, “taking medication or supplement”, and “money spent on PA activities” as predictor variables (*χ*2 (15) = 367.0, *p* < 0.001, Goodness of fit Pearson *χ*2 (6642) = 6679.3, *p* = 0.37). Comparison of the models suggested significant improvement when predictors were added (*χ*2 = 367.0 – 149.5 = 217.5, df = 15 – 3 = 12, *χ*2 (12) = 217.5 *p* < 0.001).

### *Sex and degree of education as moderating variables for cessation*

#### Sex

Multinomial logistic regression with “sex” as moderating variable was calculated with cessation as dependent and “age”, “degree of education”, “primary healthcare utilization”, “social support”, “moods”, and “QoL” as predictor variables. The overall the model was significant (*χ*2 (105) = 387.4, *p* < 0.001) with good data fit (Pearson *χ*2 (6552) = 6711.0, *p* = 0.083). Of the predictor variables, significant interaction was observed for “sex” * “taking medication or supplements” (*χ*2 (6) = 24.7, *p* < 0.001), “sex” * “QoL Environment” (*χ*2 (6) = 58.0, *p* < 0.001), and “sex” * “family discouragement for diet” (*χ*2 (6) = 19.4, *p* = 0.004).

Being woman was associated with lower perceived family support for diet changes within all groups (very early, early, and late cessation groups). For women lower likelihood of taking medication or supplements was observed in very early cessation group (group1) and for men in early cessation group (group 2). Further, only for men in very early cessation group (group 1) lower perceived environmental QoL was observed and only for women in early cessation group (group 2). Results and parameter estimates for variables associated with different group memberships are summarized in Table 4.

#### Degree of education

Multinomial logistic regression was calculated with “degree of education” as moderating variable, “cessation” as a dependent variable, and “age”, “healthcare utilization”, “social support”, “moods”, and “QoL” as predictor variables. The overall model was significant (*χ*2 (267) = 590.7, *p* < 0.001), but without good data fit (Pearson *χ*2 (6390) = 6592.5, *p* = 0.038). Of the predictor variables, significant interaction was observed for different interactive combinations: “degree of education” * “taking medication or supplements” (*χ*2 (15) = 33.3, *p* = 0.004), “degree of education” * “QoL environment” (*χ*2 (15) = 65.8, *p* < 0.001), and “degree of education” * “family discouragement for diet” (*χ*2 (15) = 38.2, *p* = 0.001).

For all groups (very early, early, and late cessation groups), a university degree was associated with lower perceived family support for diet changes. Further, university degree was associated with lower likelihood of taking medication or supplements among those in very early cessation group (group 1). Lower perceived environmental QoL was associated with university degree, but only for early cessation group (group 2). Results and parameter estimates for variables associated with different group memberships are summarized in Table 4.

### Predictors of chronic stress

Multinomial logistic regression with “chronic stress” as an outcome variable and “age”, “degree of education”, “healthcare utilization”, “social support”, and “QoL” as predictor variables indicated that overall model significance (*χ*2 (66) = 1729.6, *p* < 0.001) but without good data fit (Pearson *χ*2 (6591) = 44,469.8, *p* < 0.001). Of the predictor variables “QoL physical health” (*χ*2 (3) = 21.3, *p* < 0.001), “QoL phycological health” (*χ*2 (3) = 54.6, *p* < 0.001), “QoL environment” (*χ*2 (3) = 85.2, *p* < 0.001), “mood states” (*χ*2 (3) = 447.3, *p* < 0.001), and “sex” (*χ*2 (3) = 11.8, *p* = 0.008) were significant predictors.

Medium–low, medium–high, and high stress groups were associated with lower QoL for both psychological health and environment, as well as higher mood disturbances. Furthermore, medium–high and high stress were both associated with woman sex, with high stress being also associated with lower reported physical health QoL. Results and parameter estimates for variables associated with different group memberships are summarized in Table 5.

**Table 5:** Parameter estimates for significant predictor variables for chronic stress compared to low chronic stress

	**β**	**Standard error**	**Wald**	**Df**	**Significance**	**Ex*p*(β)**
VARIABLES ASSOCIATED WITH STRESS						
Low-medium chronic stress						
QoL Psychological health	-.17	.05	13.22	1	*p* < .001	.85
QoL Environment	-.22	.04	25.75	1	*p* < .001	.80
Mood disturbance (POMS)	.04	.01	64.74	1	*p* < .001	1.04
High-medium chronic stress						
QoL Psychological health	-.33	.05	38.24	1	*p* < .001	.72
QoL Environment	-.41	.05	68.07	1	*p* < .001	.67
Mood disturbance (POMS)	.07	.01	148.11	1	*p* < .001	1.07
Sex—Women	.50	.16	9.17	1	*P* = .002	1.64
High chronic stress						
QoL Physical health	-.19	.05	12.36	1	*p* < .001	.83
QoL Psychological health	-.43	.06	46.52	1	*p* < .001	.65
QoL Environment	-.47	.06	64.81	1	*p* < .001	.63
Mood disturbance (POMS)	.10	.01	269.08	1	*p* < .001	1.10
Sex—Women	.66	.21	9.49	1	*p* = .002	1.93
SEX AS MODERATOR OF STRESS						
Low-medium chronic stress						
QoL Psychological health – Women	-.20	.06	12.85	1	*p* < .001	.82
QoL Environment –						
Women	-.18	.05	11.51	1	*p* = .001	84
Men	-.33	.08	18.66	1	*p* < .001	.72
*Between levels no significant difference*						
Mood disturbances –						
Women	.04	.01	31.78	1	*p* < .001	1.04
Men	.05	.01	32.19	1	*p* < .001	1.05
*Between levels no significant difference*						
High-medium chronic stress						
QoL Psychological health –						
Women	-.33	.06	27.14	1	*p* < .001	.72
Men	-.32	.10	10.82	1	*p* = .001	.73
*Between levels no significant difference*						
QoL Environment –						
Women	-.38	.06	41.24	1	*p* < .001	.69
Men	-.46	.09	25.53	1	*p* < .001	.63
*Between levels no significant difference*						
Mood disturbance –						
Women	.06	.01	75.38	1	*p* < .001	1.06
Men	.08	.01	72.97	1	*p* < .001	1.08
*Between levels no significant difference*						
High chronic stress						
QoL Physical health -						
Women	-.24	.06	14.20	1	*p* < .001	.78
QoL Psychological Health –						
Women	-.40	.07	28.57	1	*p* < .001	.67
Men	-.56	.12	20.32	1	*p* < .001	.57
*Between levels no significant difference*						
QoL Environment –						
Women	-.40	.07	33.45	1	*p* < .001	.67
Men	-.67	.11	35.13	1	*p* < .001	.51
*Between levels no significant difference*						
Mood disturbance –						
Women	.09	.01	156.68	1	*p* < .001	1.10
Men	.11	.01	105.36	1	*p* < .001	1.11
*Between levels no significant difference*						
DEGREE OF EDUCATION AS MODERATOR OF STRESS						
Low-medium chronic stress						
QoL Psychological health – University education	.25	.08	9.35	1	*p* = .002	.78
QoL Environment –						
Secondary vocational education	-.70	.12	10.32	1	*p* = .001	.69
Higher vocational education	-.45	.12	14.73	1	*p* < .001	.64
*Between levels no significant differences*						
Mood disturbance -						
Up to secondary education	.05	.01	12.56	1	*p* < .001	1.05
Secondary vocational education	.03	.01	7.18	1	*p* = .007	1.03
Higher vocational education	.04	.01	11.75	1	*p* = .001	1.05
University education	.05	.01	27.36	1	*p* < .001	1.05
*Between levels of up to secondary and secondary vocational z-score* = *2.43 significant*						
High-medium chronic stress						
QoL Psychological health -						
Secondary vocational education	-.362	.13	8.02	1	*p* = .005	.70
Higher vocational education	-.408	.14	8.44	1	*p* = .004	.67
University education	-.398	.09	19.45	1	*p* < .001	.67
*Between levels no significant differences*						
QoL Environment –						
Secondary vocational education	-.49	.12	15.47	1	*p* < .001	.61
Higher vocational education	-.55	.14	15.33	1	*p* < .001	.58
University education	-.35	.08	18.74	1	*p* < .001	.71
Other	-.69	.17	15.55	1	*p* < .001	.50
*Between levels no significant differences*						
Mood disturbance –						
Up to secondary education	.07	.01	24.53	1	*p* < .001	1.07
Secondary vocational education	.05	.01	18.37	1	*p* < .001	1.05
Higher vocational education	.07	.01	25.59	1	*p* < .001	1.07
University education	.08	.01	62.58	1	*p* < .001	1.08
Other	.06	.01	18.07	1	*p* < .001	1.07
*Between levels no significant differences*						
High chronic stress						
QoL Psychological health -						
Secondary vocational education	-.46	.15	9.80	1	*p* = .002	.63
Higher vocational education	-.58	.19	8.93	1	*p* = .003	.56
University education	-.49	.11	20.39	1	*p* < .000	.61
*Between levels no significant differences*						
QoL Environment –						
Up to secondary education	-.36	.14	63.6	1	*p* = .012	.70
Secondary vocational education	-.53	.15	13.04	1	*p* < .001	.59
Higher vocational education	-.83	.19	19.49	1	*p* < .001	.43
University education	-.37	.09	14.93	1	*p* < .001	.69
Other	-.65	.21	9.83	1	*p* = .002	.52
*Between levels no significant differences*						
Mood disturbance –						
Up to secondary education	.10	.01	46.03	1	*p* < .001	1.11
Secondary vocational education	.07	.01	26.55	1	*p* < .001	1.07
Higher vocational education	.10	.02	39.55	1	*p* < .001	1.11
University education	.12	.01	116.33	1	*p* < .001	1.13
Other	.10	.02	33.15	1	*p* < .001	1.10
*Between levels of up to secondary and secondary vocational z-score* = *2.42 significant*						

### Chronic stress—Independence of the significant predictors from BMI

Two logistic regression models were calculated with chronic stress as the dependent variable. The first model was calculated with “BMI” as the predictor variable (*χ*2 (3) = 61.18, *p* < 0.001, Goodness of fit Pearson *χ*2 (6582) = 6578.85.0, *p* = 0.51). The second model was calculated with “BMI”, “QoL psychological health”, “QoL physical health”, “QoL environment”, “mood disturbances”, and “sex” as predictor variables (*χ*2 (18) = 1638.42, *p* < 0.001, Goodness of fit Pearson *χ*2 (6639) = 17,229.36, *p* < 0.001). Comparison of the models suggested significant improvement between the models when predictors were added (*χ*2 = 1638.42 – 61.18 = 1557.24, df = 18 – 3 = 15, *χ*2 (15) = 1557.24 *p* < 0.001).

### Sex and degree of education as moderating variables for chronic stress

#### Sex

Multinomial logistic regression with sex as moderating variable was calculated with “age”, “degree of education”, “primary healthcare utilization”, “social support”, and “QoL”. The overall the model was significant (*χ*2 (105) = 1756.7, *p* < 0.001) but without good data fit (Pearson *χ*2 (6552) = 35,745.11 *p* < 0.001). Of the predictor variables, significant interaction with sex was observed for “QoL physical health” (*χ*2 (6) = 28.1, *p* < 0.001), “QoL psychological health” (*χ*2 (6) = 58.1, *p* < 0.001), “QoL environment” (*χ*2 (6) = 92.5, *p* < 0.001), and “mood disturbances” (*χ*2 (6) = 453.0, *p* < 0.001).

Being man or woman was found to moderate the associations for low-medium and high stress. For low-medium stress group lower psychological health QoL and for high stress group lower physical health QoL were associated with women but not with men. Results and parameter estimates for variables associated with different group memberships are summarized in Table 5.

#### Degree of education

Multinomial logistic regression with “degree of education” as moderating variable was calculated. The overall model was significant (*χ*2 (267) = 1962.9, *p* < 0.001) but Goodness-of-Fit test did not indicate good data fit (Pearson χ2 (6390) = 12,240.0 *p* < 0.001). Of the predictor variables, significant interaction with “degree of education” was observed for “QoL physical health” (χ2 (15) = 33.0, *p* = 0.005), “QoL psychological health” (χ2 (15) = 67.2, *p* < 0.001), “QoL environment” (χ2 (15) = 100.2, *p* < 0.001), and “mood states” (χ2 (15) = 440.1, *p* < 0.001).

Also, degree of education moderated the association for low-medium and high stress groups. For the low-medium stress group university degree was associated with lower psychological health QoL. For both low-medium and high stress groups, those with up to a secondary degree of education reported fewer mood disturbances than those with higher degree of education. Results and parameter estimates for variables associated with different group memberships are summarized in Table 5.

## Discussion

Achieving weight-loss and weight-loss maintenance, key components of T2D-prevention, can be very challenging even when supportive behavioral interventions are offered [17, 18]. In the present study, variables and pathways, i.e. interactions between intervention inputs, individuals, and context variables [19, 21, 22], associated with premature intervention cessation and chronic stress at the start of an intervention were examined [16, 36]. Our results supported the notion that successful intervention completion is a complex and dynamic process, relying on interactions between intervention inputs and personal factors [19, 21, 22]. Findings indicated that pathways between QoL, social support, primary care utilization, mood and chronic stress as well as cessation were moderated by both sex and degree of education, a prominent and significant dimension of SES.

As expected based on the previous research, overall lower QoL was associated with both intervention cessation and chronic stress [14, 15]. Although only lower environmental QoL was associated with cessation [16, 28, 29], higher chronic stress was more broadly associated with lower QoL [8, 9]. It is not clear why only environmental QoL, which according to WHO [64] encompasses aspects such as safety, access to medical services, availability of resources, and opportunities for skills acquisition was associated with cessation. Furthermore, the result is difficult to interpret as lower environmental QoL was associated with very early intervention cessation especially with men and with early cessation especially for women and those with a university degree, which, in itself, was associated with higher QoL [47].

In accordance with previous research [29, 71], lack of family support was associated with earlier intervention cessation especially for women in this study. Furthermore, SES, represented here as degree of education [5, 56], was found to moderate between intervention cessation and social support, especially lack of family support for diet changes. Lack of family support was associated particularly with university degree. While in former studies being a man who had reached only a lower degree of education had been associated with less favorable intervention outcomes [18, 38, 45], in this study women with higher degrees of education were at risk of poorer intervention outcomes. Although degree of education and sex can moderate relationship between intervention outcomes [38, 41, 50], in the present study we observed that higher degrees of education bear overall a risk for non-completing the PREVIEW intervention. Combined with the observation that in PREVIEW intervention single parents were least likely to achieve weight-loss, our results highlighted the lack of resources such as time as a factor for less favorable outcomes especially for university educated women with family responsibilities.

As mood disturbances are closely associated with stress [52], association between the variables was expected. Despite previous research indicating sex as a potential moderating variable [42], in this study sex was not found to moderate the relationship between mood disturbances and chronic stress, thereby adding to the inconclusive body of literature examining mood disturbances in association with intervention cessation [22]. Nonetheless, it could be postulated that the lack of association may be attributable to participant selection [58], given that those with major mental health difficulties were excluded.

While it was hypothesized that higher primary care utilization prior to intervention enrolment [36] would be associated with cessation and chronic stress, only higher non-usage of medication or supplements was associated with very early intervention cessation, especially for women and those with university education. This result emphasized further the complexity of pathways leading to unsuccessful intervention completion [27, 72]. As hesitancy about lifestyle changes and their necessity may hinder participation [24–26], participants without comorbidities requiring medication may perceive themselves at lower risk of adverse consequences from prediabetes, thus leading to a higher risk of cessation.

Elevated stress is considered to lower the likelihood of successful weight-loss and weight-loss maintenance in lifestyle change interventions [18, 36]. Participants living with children and those economically not active due to, e.g. caring responsibilities, were least likely to report low chronic stress at the start of the intervention. Overall, as expected, higher chronic stress was associated with lower psychological QoL and higher mood disturbances, indicating that factors such as low self-esteem, negative feelings, and negative body image may have amplified chronic stress [18]. Although men from lower socio-economic background have been reported to be particularly vulnerable to experience stress [31], in here, especially women reported medium–high and high chronic stress.

From the results, it was notable that high chronic stress was associated with significantly lower physical health QoL especially for women. Physical health QoL encompasses concepts such as energy and fatigue, sleep and rest, and mobility. As stress has been associated with physical inactivity [36] and the risk of weight gain [32, 55], the current results suggested that lower physical health QoL at the start of the intervention may predispose especially women to higher chronic stress and thus to suboptimal weight-loss outcomes. Further, although lower SES has been associated with increased stress and consequently to worse weight-loss outcomes [44–46], in the present study there was only the results indicated only limited influence of SES to chronic stress.

Targeted strategies are required to improve T2D-prevention especially in primary care settings [37]. Success of behavior change interventions in T2D-prevention is based on complex interactions between participants and intervention [26, 72]. Both intervention cessation and chronic stress at the start of the intervention are important determinants of successful weight-loss and weight-loss maintenance [26, 28, 45]. Most differences were found between intervention completers and those who discontinued the intervention very early or early, and participants reporting high or low chronic stress. Lower environment al QoL and lack of family support for diet changes emerged as important predictors for cessation with women and those with higher SES especially affected. In turn, high chronic stress was predicted by higher mood disturbances and lower QoL for psychological and physical health, with, yet again, women more affected. Finally, the analyses indicated that the identified predictor variables were independent of participant BMI [25], further highlighting the complexity of pathways that healthcare professionals need to consider in planning and delivering T2D-prevention interventions. For public health promotion, the results indicate that intervention developers and practitioners engaged in T2D-prevention need to consider how flexible intervention elements could be incorporated into the design and delivery to ensure better fit of varied participant`s needs.

There are numerous strengths associated with the study, particularly the large sample size. Nonetheless, the study is not without limitations. Specifically, participants were divided in the groups retrospectively, and it is recognized that different group divisions could have influenced the results. While logistic regression as an analysis method places few limitations on the data, stress and mood states were, as expected, correlated (*r* = .07). Furthermore, associations between the outcome variables of chronic stress and cessation were not examined and additional work at this area would be needed. Also, degree of education was used as a measurement of SES [5, 49, 56], and it can argued that other measurements e.g. incorporating income in to measurement of SES might have been more appropriate [6], although not unproblematic in international research. In addition, no adjustments were made regarding different access to university education between countries. Number of predictor variables included in the analyses was also restricted, limiting ability to test different pathways. Finally, the interpretation of the results should be done carefully, as due to large number of participants, even small differences could produce statistically significant associations.

## Conclusions

Despite of the study limitations, the results contribute to the knowledge of factors and pathways associated with unsuccessful completion of preventive T2D interventions. Overall, lack of initial family support for diet changes and lower QoL appeared significant to hinder successful intervention completion, with women and those with higher degree of education especially impacted. The findings may reflect difficulties that are particularly faced by women to achieve and maintain new behaviors while dealing with demands of family and work. Family and work commitments may also lead to increased stress, which in itself can be counterproductive for successful weight-loss. Different aspects of QoL predicted cessation and stress, indicating that existing stressors such as lack of resources or negative feelings may lead participants to struggle with successful intervention completion. Whilst it may well be unrealistic to expect all participants to successfully complete an intervention, we feel that healthcare professionals involved in design and delivery of T2D-prevention interventions may need to take into consideration not only participant characteristics but also their life situation. This would mean developing strategies that allow flexible intervention designs and implementation features, to, for example, enable involvement of families or strengthen individuals’ skills to successfully navigate the process of behavior change despite demands of family and work life.

## Data Availability

Data not available due to ethical/legal/commercial restrictions. Due to the nature of this research, participants of this study did not agree for their data to be shared publicly, so supporting data is not available. With reasonable request from the reviewers and subject to written agreement from all study sites and study authors, deidentified data used in this study may be made available for the reviewers.
